# Transanal total mesorectal excision: single center study on risk factors for major complications

**DOI:** 10.3389/fonc.2023.1277979

**Published:** 2023-10-19

**Authors:** Zhiwen Xu, Jingtao Zhu, Haoyu Bai, Liangbin Xiao, Tinghao Wang, Hexin Lin, Qingqi Hong, Jun You

**Affiliations:** ^1^ School of Medicine, Xiamen University, Xiamen, China; ^2^ School of Clinical Medicine, Fujian Medical University, Fuzhou, China; ^3^ Department of Gastrointestinal Oncology Surgery, The First Affiliated Hospital of Xiamen University, Xiamen, China

**Keywords:** total mesorectal excision, transanal, rectal cancer, complications, risk factors

## Abstract

**Purpose:**

Transanal total mesorectal excision (TaTME) as a novel surgical approach for mid and low rectal cancer has gained significant research interest in recent years. The main objective of this study is to identify the risk factors associated with major complications after TaTME and evaluate the perioperative clinical outcomes.

**Methods:**

A retrospective analysis was performed on the clinical data of patients with mid-to-low rectal cancer who underwent TaTME surgery and were admitted to the First Affiliated Hospital of Xiamen University from January 2018 to May 2023. Univariate and multivariate regression methods were employed to analyze the risk factors influencing the occurrence of major complications (Clavien-Dindo III-V).

**Results:**

This study included a total of 179 eligible cases, with no perioperative deaths. The overall incidence of early complications was 25.1%, with a rate of 10.1% for mild complications and 15.0% for major complications. The postoperative anastomotic leakage rate within 30 days was 6.7%. Multivariate analysis demonstrated that male (P=0.030), pathological T ≥ 3 (P=0.018) and manual anastomosis (P=0.009) were independent risk factors for the development of major complications after surgery.

**Conclusion:**

In this study, the incidence of early complications and anastomotic leakage rate in TaTME were both relatively low. Male, pathological T stage ≥ 3 and manual anastomosis were independent risk factors for the occurrence of major complications in a cohort of patients with mid and low rectal cancer undergoing TaTME.

## Introduction

In 1982, British scholar Heald proposed total mesorectal excision (TME) as a surgical approach for rectal cancer (1). After several decades of research, clinical application, and validation, TME has become the standard procedure for surgical treatment of rectal cancer (2). Complete mesorectal excision is an important criterion for high-quality TME, and compared to procedures that do not achieve complete TME, it effectively reduces the risk of local tumor recurrence and distant metastasis. This achievement has gained wide recognition in the academic community (3).

In clinical practice, both laparoscopic and open abdominal TME procedures face challenges in achieving high-quality surgical outcomes for difficult pelvic cases, such as those involving obesity, enlarged prostate, thickened mesorectum, and narrow pelvic space. The emergence of transanal total mesorectal excision (TaTME) has partially addressed these limitations of abdominal TME (4).

TaTME has emerged as an innovative surgical approach aimed at preserving anal function in patients with mid and low rectal cancer, and has gained significant attention in the field of clinical research in recent years. Despite its growing popularity, TaTME remains relatively new, and there is a scarcity of high-quality clinical evidence to support its widespread adoption. TaTME is considered a technically demanding procedure, requiring specialized skills and expertise. Controversies persist regarding several critical aspects of TaTME, including its safety profile, perioperative outcomes, long-term survival prognoses, and functional outcomes postoperatively (5, 6).

Since the implementation of this technique in our center in 2017, we have gradually gained technical maturity after overcoming the learning curve from the initial stage. Currently, our center has accumulated substantial experience in TaTME. The primary aims of this study are to identify the risk factors for major complications and to investigate and analyze the perioperative efficacy of TaTME based on data from our center. We hope that this study will provide valuable insights for the further development and clinical application of this technique.

## Materials and methods

### Study design

A retrospective analysis was conducted on clinical and pathological data of patients with mid or low rectal cancer who underwent TaTME at the First Affiliated Hospital of Xiamen University from January 2018 to May 2023.

Inclusion criteria: (1) Patients with mid or low rectal cancer who underwent TaTME with or without intersphincteric resection (ISR). Exclusion criteria: (1) Patients with imaging indicates preoperative metastasis. (2) Patients requiring multi-organ resection. (3) Patients with missing clinical or pathological data. The study was approved by the Ethics Committee at the First Affiliated Hospital of Xiamen University.

### Surgical technique

**Figure 1:**
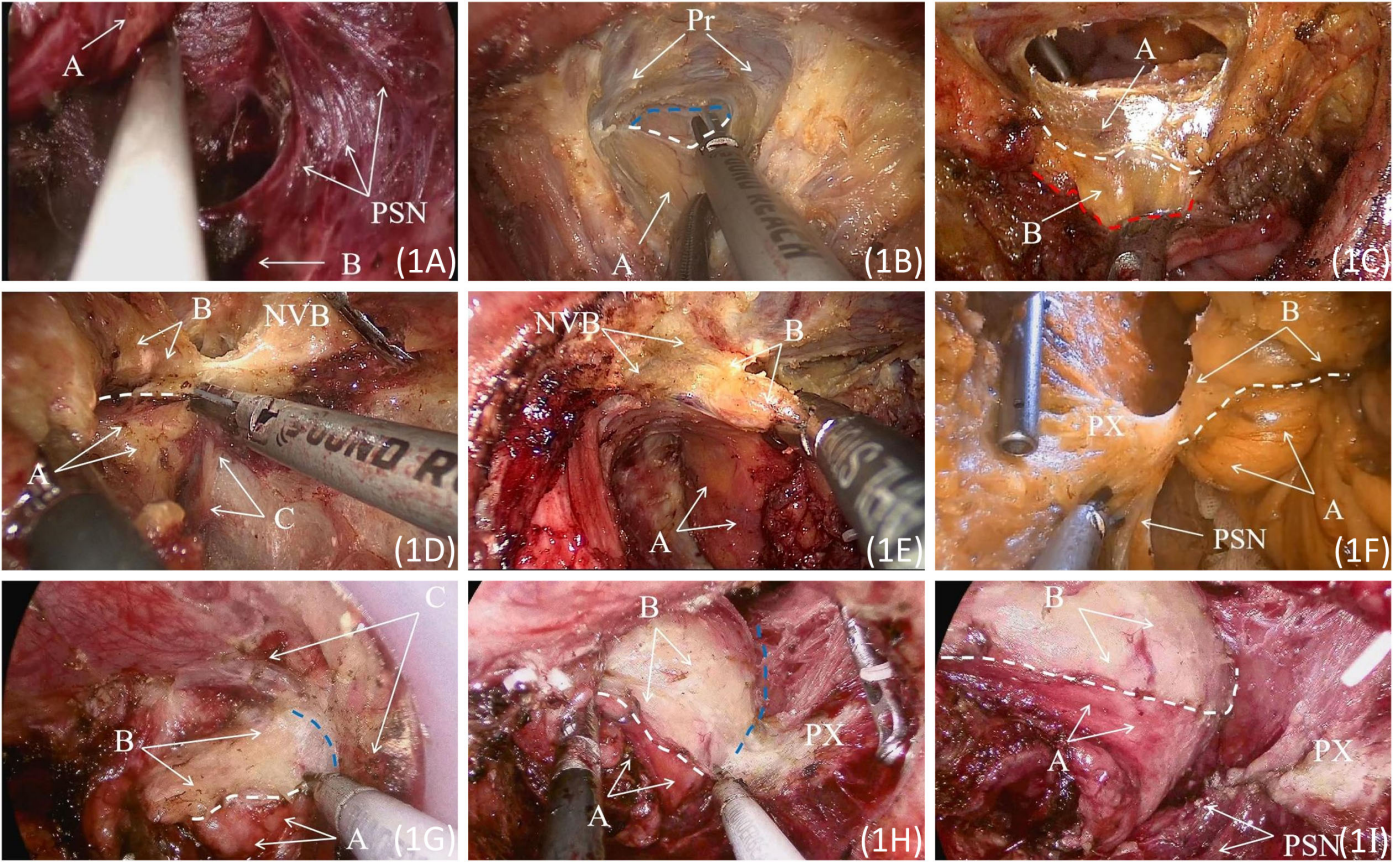
**(A)** Exposed pelvic splanchnic nerves. A: Rectal posterior fascia; B: The presacral fascia continuous with the levator ani fascia. **(B)** The Denonvilliers’ fascia is incised at the base of the seminal vesicle. A: Fascia Propria of The Rectum. The white dashed line represents the posterior end of Denonvilliers’ fascia during surgery; the blue dashed line indicates the retroprostatic space. **(C)** The rectal and abdominal groups converge at the level of the peritoneal reflection. A: Denonvilliers Fascia; B: Fascia Propria of The Rectum. The white dashed line represents the posterior end of Denonvilliers’ fascia; the red dashed line indicates the edge of the rectal fascia propria. **(D)** Incising the anterior lateral fascia of the pre-hypogastric nerve. A: Pre-hypogastric Nerve Fascia; B: Fascia Propria of The Rectum; C: Left S4 pelvic splanchnic nerves. The white dashed line represents the intraoperative margin of the pre-hypogastric nerve fascia. **(E)** Separating the anterior lateral space of the rectum. A: Pre-hypogastric Nerve Fascia; B: Fascia Propria of The Rectum; C: Left S4 pelvic splanchnic nerves. **(F)**Incising the pre-hypogastric nerve fascia posteriorly, separating the posterior lateral space of the rectum. A: Pre-hypogastric Nerve Fascia; B: Fascia Propria of The Rectum. The white dashed line represents the intraoperative margin of the pre-hypogastric nerve fascia. **(G)** Separating the lateral space of the rectum in a direction from the abdominal side to the posterior side. A: Pre-hypogastric Nerve Fascia; B: Fascia Propria of The Rectum; C: Denonvilliers’ fascia and pre-hypogastric nerve fascia transition zone. The white dashed line represents the intraoperative margin of the pre-hypogastric nerve fascia, and the blue dashed line indicates the lateral space of the anterior rectum. **(H)** Separating the lateral space of the rectum in a direction from the abdominal side to the posterior side. A: Pre-hypogastric Nerve Fascia; B: Fascia Propria of The Rectum. The white dashed line represents the intraoperative margin of the pre-hypogastric nerve fascia, and the blue dashed line indicates the lateral space of the rectal side. **(I)** The dissected pelvic splanchnic nerves and pelvic plexus are gently pushed outward. A: Pre-hypogastric Nerve Fascia; B: Fascia Propria of The Rectum. The white dashed line represents the intraoperative margin of the pre-hypogastric nerve fascia. PSN, Pelvic Splanchnic Nerves; Pr, Prostate. NVB, Neurovascular Bundle; PX, Pelvic Plexus.

In this study, TaTME refers to laparoscopic-assisted TaTME. The surgery was performed simultaneously by two groups of surgeons, one entering from the abdominal direction and the other from the anal direction, collaborating to complete the procedure. The surgical illustrations are shown in **Figures 1A–I**. The abdominal portion is performed under laparoscopic guidance, involving the preservation of the left colic artery and D3 lymph node dissection. Routine dissection includes clearing the No. 253 lymph nodes around the inferior mesenteric artery (IMA) (7, 8). The dissection extends anteriorly to the level of the seminal vesicles and posteriorly to the level of the sacral fascia.

During the transanal phase, the LoneStar retractor was used to expose the anus, and the STAR-PORT endoscopic platform along with insufflation facilitated visualization. A purse-string suture was placed approximately 2 cm proximal to the distal tumor margin to prevent potential tumor cell shedding. Subsequently, at a distance of 1cm from the purse-string suture site, a low-energy setting electrocautery was employed to create a circumferential marking on the rectal wall, followed by a full-thickness incision of the rectal wall. For patients with mid-to-low rectal cancer, the location of the enterotomy was typically at the termination of the mesorectum near the anorectal ring. The separation of the rectal lateral spaces proceeded in the order of posterior, anterior, anterior-lateral, and lateral-posterior directions, according to the distribution and characteristics of the autonomous nerves.

During the dissection of the posterior rectal space, the rectal wall’s full-thickness incision should be immediately followed by separation along the surface of the levator ani fascia until the levator ani’s apex is reached. At this point, the fusion fascia of the posterior rectal space is identified. The anterior rectal space lies between Denonvilliers’ fascia and the fascia propria of the rectum. Incising the anterior rectal wall at the level of the levator ani hiatus allows access to the anterior rectal space. As there are no nerves or blood vessels within the anterior rectal space, the surgeon can proceed directly laterally to dissect the anterior rectal space towards the head until reaching the peritoneal reflection. Alternatively, the Denonvilliers Fascia can be incised at the base of the seminal vesicle to meet with another surgical team in the retroprostatic space located behind the prostate. Injury to the neurovascular bundle (NVB) in the prostatic region is one of the causes of postoperative urinary and sexual dysfunction in rectal cancer surgery. During the separation of the retroprostatic space, it is important to closely adhere to the surface of the Fascia Propria of The Rectum to protect the NVB. The use of an ultrasonic scalpel in a low-energy mode or the application of vascular clips followed by division of the rectal vascular branches assists in preserving the NVB in the prostatic region.

When dissecting the anterior lateral rectal space, the pre-hypogastric nerve fascia is incised. The separation then proceeds bilaterally, and finally, the fascia propria of the rectum is detached from the lateral side to the posterior side. Resistance is encountered at the fusion site between the pre-hypogastric nerve fascia and the fascia propria of the rectum during lateral posterior rectal space dissection. At this point, the pre-hypogastric nerve fascia can be incised, and the pelvic splanchnic nerves can be gently pushed laterally, creating a communication between the lateral and posterior rectal spaces. The anterior lateral rectal space is located between Denonvilliers’ fascia and the fascia propria of the rectum. During its dissection, close adherence to the fascia propria of the rectum’s surface is essential for the protection of the NVB in the prostate region and the internal anal sphincter nerves. The transanal dissection progresses until reaching the level of the rectosacral fascia and meeting with the abdominal group. For descriptions of other surgical techniques, including the intersphincteric resection section, reference can be made to our team’s previous studies (9). All surgical procedures adhere to the basic principles outlined in relevant clinical guidelines (10).

### Endpoints and definitions

The primary endpoint of this study was to identify risk factors associated with major complications within 30 days after surgery. Secondary endpoints included the incidence of anastomotic leakage, postoperative pathological findings, surgical duration, intraoperative blood loss, postoperative hospital stay, and duration of postoperative abdominal drainage tube placement.

Evaluation criteria: Early complications are defined as complications occurring within 30 days after surgery, while late complications are defined as complications occurring more than 30 days after surgery. Mild complications are classified as Grade I-II, whereas major complications are categorized as Grade III-V, following established medical standards and classification systems.

Early complications are graded using the Clavien-Dindo (CD) classification as follows: Grade I: Complications that do not require medication treatment and are managed with routine measures such as antipyretics or analgesics; Grade II: Complications that require treatment beyond the measures mentioned above; Grade III: Complications requiring surgical or endoscopic intervention; Grade IV: Severe complications that are life-threatening, such as cerebral hemorrhage; Grade V: Patient death (11).

The diagnosis and severity grading of anastomotic leakage will follow the 2010 criteria established by the International Study Group of Rectal Cancer (ISREC). The diagnostic criteria for anastomotic leakage are as follows: (1) Drainage of intestinal contents from the presacral drain or abdominal incision. (2) Visualization of contrast agent flowing out from the drainage tube during gastrointestinal contrast imaging. (3) CT scan revealing disruption of the intestinal wall or presence of gas or fluid around the anastomosis. (4) Confirmation of anastomotic disruption during repeat surgery (12).

The grading of anastomotic leakage is as follows: Grade A indicates no need for invasive intervention. Grade B requires invasive intervention but does not necessitate surgical treatment. Grade C requires surgical intervention.

### Follow-up

Postoperative follow-up is conducted by specialized personnel through various methods, including telephone interviews, outpatient visits, and inpatient reevaluations. Generally, within the first 2 years after surgery, outpatient visits are scheduled every 3 months. During the period of 2 to 5 years post-surgery, patients are followed up every 6 months, and after 5 years from the surgery, they are reevaluated annually.

### Statistical analyses

Data analysis was performed using SPSS 26.0 statistical software. Normally distributed continuous data are presented as mean ± standard deviation (SD), while skewed distributed continuous data are presented as median (range). Categorical data are presented as frequencies and percentages. Univariate and multivariate analyses were performed to identify possible risk factors associated with major complications. Univariate analysis comparing categorical variables was performed using the Pearson X^2^ test, and continuous variables were analyzed using Mann-Whitney U test. Variables with a P-value of ≤ 0.2 on univariate analysis were included in the multivariate analysis to identify independent predictors of major complications. Multivariate analysis was subsequently performed using Logistic regression. In the multivariate analysis, variables with a P-value of < 0.05 were considered statistically significant.

## Results

### Perioperative results

**Figure 2:**
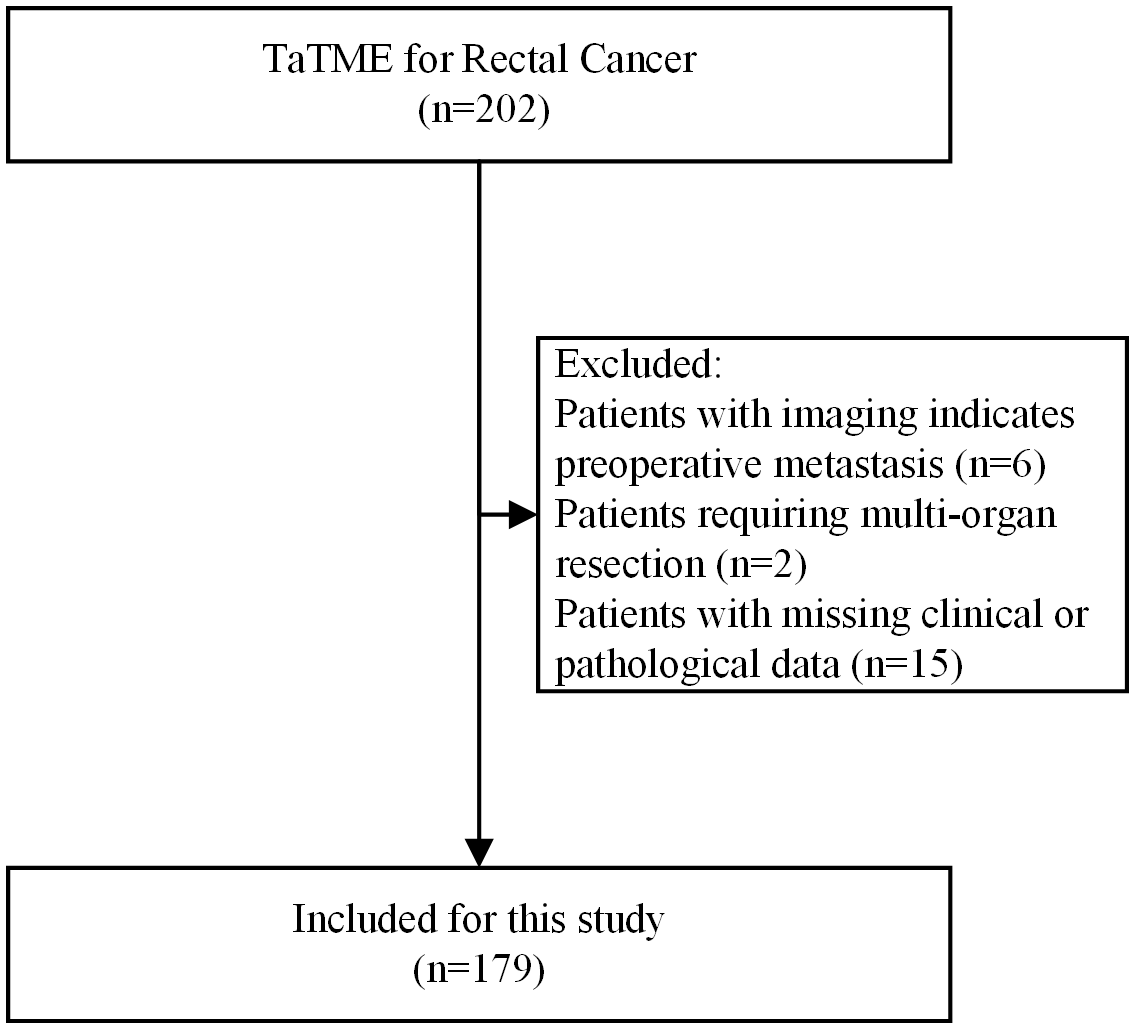
CONSORT diagram of this study.

Based on the inclusion and exclusion criteria, a total of 179 cases that met the criteria were included in this study. The consort diagram of this study are shown in **Figure 2** and baseline characteristics are shown in **Table 1**. All patients successfully underwent surgery according to the preoperative plan. Among them, 44 (24.6%) patients underwent combined ISR. There were no deaths during the perioperative period. All surgeries were performed simultaneously by two surgical groups, one group through the abdomen and the other through the anus. The median operative time was 228.07 (110–395) minutes, median intraoperative blood loss was 55.47 (20–500) ml, median postoperative hospital stay was 11.14 (5–40) days, time to first soft diet was 5.32 (2–23) days, median duration of gastric tube placement was 1.37 (0–11) days, and median duration of abdominal drainage tube placement was 8.82 (4–37) days.

**Table 1:** Demographic features and clinical outcomes of 179 patients who underwent TaTME.

Characteristic	Data
Gender, n (%)	
Female	61 (34.1%)
Male	118 (65.9%)
Age, mean ± SD (year)	59.66 ± 10.784
BMI, mean ± SD (kg/m^2^)	23.14 ± 3.092
Height (cm)	163.44 ± 7.635
Weight (kg)	61.97 ± 10.117
Smoker, n (%)	26 (14.5%)
Hypertension, n (%)	42 (23.5%)
Diabetic, n (%)	27 (15.1%)
History of surgery for benign diseases, n (%)	11 (6.2%)
Neoadjuvant therapy, n (%)	49 (27.4%)
Neoadjuvant chemotherapy, n (%)	27 (15.1%)
Combined ISR, n (%)	44 (24.6%)
Height from anal verge [cm,Median (range)]	5.06 (2–10)
Operative time [min,M(range)]	228.07 (110–395)
Intraoperative blood loss [ml, M(range)]	55.47 (20–500)
Anastomotic technique, n (%)	
Manual	45 (25.1%)
Stapled	134 (74.9%)
Ileostomy, n (%)	
No	2 (1.1%)
Yes	177 (98.9%)
Postoperative hospital stay [d,M(range)]	11.14 (5–40)
Time to first soft diet [d,M(range)]	5.32 (2–23)
Removal of abdominal drainage [d,M(range)]	8.82 (4–37)
Removal of gastric tube [d,M(range)]	1.37 (0–11)

BMI, body mass index.

TaTME, Transanal total mesorectal excision.

### Postoperative pathological outcomes

Among the 179 patients included in this study, postoperative pathological examination revealed negative circumferential margins and negative distal margins in all patients. The median tumor diameter was 3.30 (0.60-10.50) cm, with a median of 20.41 (8–57) harvested lymph nodes and a median of 1.24 (0-17.00) positive lymph nodes. Postoperative tumor staging according to the TNM classification was as follows: stage I in 76 (42.5%) cases, stage II in 45 (25.1%) cases, and stage III in 58 (32.4%) cases. Refer to **Table 2** for details.

**Table 2:** Pathologic results of 179 patients who underwent TaTME.

Characteristic	Data
Pathological T stage, n (%)	
pT1	17 (9.5%)
pT2	77 (43.0%)
pT3	67 (37.4%)
pT4	18 (10.1%)
Pathological N stage, n (%)	
pN0	121 (67.6%)
pN1	54 (30.2%)
pN2	4 (2.2%)
Pathological TNM stage, n (%)	
I	76 (42.5%)
II	45 (25.1%)
III	58 (32.4%)
Tumor size, M (range) (cm)	3.30 (0.60-10.50)
Number of lymph nodes harvested, M (range)	20.41 (8–57)
Number of positive lymph nodes, M (range)	1.24 (0-17.00)
Length between tumor and DRM, mean ± SD (cm)	2.88 ± 1.08
CRM status, n (%)	
Positive	0
Negative	179 (100%)
DRM status, n (%)	
Positive	0
Negative	179 (100%)

CRM, circumferential resection margin.

DRM, distal resection margin.

TNM, Tumor-node-metastasis.

Tumors were classified according to the American Joint Committee on Cancer (AJCC) TNM system.

TaTME, Transanal total mesorectal excision.

### Postoperative complications

In this group of patients, 45 (25.1%) cases experienced postoperative complications, including 18 (10.1%) cases classified as CD I-II, and 27 (15.0%) cases classified as CD III-IV. There were no cases classified as CD V. Twelve (6.7%) cases presented with anastomotic leakage, among which 5 (2.8%) cases were classified as grade A and were managed conservatively without specific intervention. One (0.5%) cases were classified as grade C anastomotic leakage and were successfully treated with intra-abdominal irrigation, drainage, and antibiotic therapy. There was one (0.5%) case of anastomotic bleeding, 8 (4.5%) cases of postoperative intestinal obstruction, 3 (1.7%) cases of urinary retention, 4 (2.2%) cases of pelvic infection, 14 (7.8%) cases of pulmonary infection, and 3 (1.7%) cases of pleural effusion. All complications were successfully managed with appropriate treatment. Refer to **Table 3** for details.

**Table 3:** Postoperative course of 179 patients who underwent TaTME.

Variables	N (%)
Postoperative complications	45 (25.1%)
Mild (Clavien-Dindo I-II)	18 (10.1%)
Major (Clavien-Dindo III-V)	27 (15.0%)
Anastomotic leakage	12 (6.7%)
Grade A	5 (2.8%)
Grade B	6 (3.4%)
Grade C	1 (0.5%)
Anastomotic bleeding	1 (0.5%)
Intestinal obstruction	8 (4.5%)
Urinary retention	3 (1.7%)
Abdominal or pelvic infection	4 (2.2%)
Pulmonary infection	14 (7.8%)
Pleural effusion	3 (1.7%)

TaTME, Transanal total mesorectal excision

### Risk factors for major complications

The results of the univariate analysis showed that male (P=0.002), weight ≥ 70 kg (P=0.063), smoker (0.079), neoadjuvant chemotherapy (P=0.139), Blood loss≥100mL (P=0.034), pathological T stage ≥ 3 (P=0.030) and manual anastomosis (P=0.043) were factors influencing the occurrence of CD ≥3 grade complications after surgery. Furthermore, these five variables with P < 0.2 were included in the multivariate analysis, and the results revealed that male (P=0.030), pathological T stage ≥ T (P=0.018) and manual anastomosis (P=0.009) were independent risk factors for the occurrence of CD ≥ 3 grade complications after surgery. Refer to **Table 4** for details.

**Table 4:** Univariate and multivariate analyses of risk factors associated with major postoperative complications.

		Univariate Analysis			Multivariate Analysis		
Variables	N(%)	Adjusted Odds ratio	95% Confidence Interval	P Value	Adjusted Odds ratio	95% Confidence Interval	P Value
Sex							
Female	61 (34.08%)	1					
Male	118 (65.92%)	7.930	1.811-34.724	0.002	5.989	1.262-28.432	0.024
Age (year)							
<55	56 (31.28%)	1					
≥55	123 (68.72%)	0.895	0.375-2.139	0.803			
BMI							
<25 kg/m²	133 (74.30%)	1					
≥25 kg/m²	46 (25.70%)	1.014	0.398-2.582	0.977			
Weight							
<70 kg	132 (73.74%)	1					
≥ 70 kg	47 (26.26%)	2.215	0.943-5.203	0.063	1.246	0.454-3.422	0.670
Smoker							
No	153 (85.47%)	1					
Yes	26 (14.53%)	2.450	0.914-6.566	0.079	2.151	0.707-6.543	0.177
Diabetes							
No	152 (84.92%)	1					
Yes	27 (15.08%)	1.782	0.644-4.931	0.254			
Hypertension							
No	137 (76.54%)	1					
Yes	42 (23.46%)	0.522	0.170-1.605	0.250			
History of surgery for benign diseases							
No	168 (93.85%)	1					
Yes	11 (6.15%)	2.250	0.557-9.083	0.219			
ASA classification							
I-II	150 (83.80%)	1					
III–IV	29 (16.20%)	0.883	0.281-2.777	1.000			
Neoadjuvant therapy							
No	130 (72.63%)	1					
Yes	49 (27.37%)	1.704	0.720-4.035	0.222			
Neoadjuvant chemotherapy							
No	152 (84.92%)	1					
Yes	27 (15.08%)	2.310	0.866-6.160	0.139	2.542	0.810-7.973	0.110
Operation time							
<240min	115 (62.25%)	1					
≥240min	64 (37.75%)	1.839	0.805-4.203	0.145	1.163	0.436-3.101	0.762
Blood loss							
<100mL	161 (89.94%)	1					
≥100mL	18 (10.06%)	3.333	1.130-9.835	0.034	1.743	0.501-6.065	0.383
Distance from anal verge							
>5cm	62 (34.64%)	1					
≤5cm	117 (75.36%)	0.884	0.378-2.068	0.776			
Tumor size							
<3cm	74 (41.34%)	1					
≥3cm	105 (58.66%)	0.861	0.377-1.965	0.722			
Pathological T stage							
pT 1-2	94 (52.51%)	1					
pT 3-4	85 (47.49%)	2.537	1.072-6.007	0.030	3.455	1.234-9.676	0.018
Pathological N stage							
pN 0	121 (67.60%)	1					
pN+	58 (32.40%)	1.536	0.662-3.562	0.315			
Anastomotic technique							
Manual	45 (25.14%)	1					
Stapled	134 (74.86%)	0.419	0.178-0.988	0.043	0.236	0.080-0.694	0.009
With ileostomy							
No	2 (1.12%)	1					
Yes	177 (98.88%)	0.987	0.969-1.005	1.000			

BMI, body mass index. ASA, American Society of Anesthesiologists. TaTME, Transanal total mesorectal excision.

## Discussion

Postoperative complications have always been an important factor affecting surgical safety and quality. In our center, all cases were performed by two teams simultaneously, which not only adhered to oncological principles but also reduced the technical difficulty of complete transanal surgery and shortened the operation time.

Through univariate and multivariate analysis of the major complications, we found that male, pathological T stage ≥ 3 and manual anastomosis were independent risk factors for the occurrence of major complications. In this study, the overall incidence of postoperative complications was 25.1%, with 10.1% classified as CD I-II and 15.0% classified as CD III-IV. The occurrence rate of anastomotic leakage was 6.7%, and only one case of C grade anastomotic leakage were observed, indicating a favorable perioperative safety profile of TaTME.

We found that pathological stage T3 or higher was an independent risk factor for the occurrence of major postoperative complications. For patients with advanced stage disease, deeper tumor infiltration into the intestinal wall inevitably leads to greater surgical complexity, potentially increasing the risk of postoperative complications.

In this study, we also found that male was an independent risk factor for the occurrence of major postoperative complications in rectal cancer patients. This may be due to the fact that males generally have a narrower pelvis compared to females, leading to increased surgical complexity and an increased risk of postoperative complications.

Postoperative complications primarily focused on pulmonary complications, with pulmonary infection being the most common, but no pulmonary-related CD III or IV complications were observed. This is likely due to factors such as advanced age in the majority of patients, highlighting the necessity of preoperative active pulmonary function exercises. In this study, it was also found that 4.5% of patients developed postoperative intestinal obstruction. Encouraging early mobilization in patients who can tolerate it may further reduce the incidence rate.

According to the Chinese CTRC database, the reported data from 2022 showed a postoperative overall complication rate of 15.4% for TaTME, which did not show a significant difference compared to traditional laparoscopic TME (13). In a study including 100 TaTME patients, the postoperative complication rate was 32% (14). Similarly, Caycedo-Marulanda et al. found a postoperative complication rate of 34% in their study (15). In a study on complications of TaTME, the early postoperative complication rate was 38.6% (16). In another comparative study between TaTME and laparoscopic TME, the TaTME group had a significantly higher early postoperative complication rate of 37.1%, which was significantly higher than the rate of 21.8% in the laparoscopic TME group (17).

The above-mentioned large-scale database studies have all shown a higher incidence of complications during the perioperative period in TaTME. However, further analysis reveals variations in the proportion of major complications and mild complications. In a study that included 767 consecutive cases, the incidence of CD ≥III complications was only 12.5% (18). In another study by Marta Penna et al., which included 720 TaTME cases, the overall incidence of postoperative complications was 33.1%, with a rate of 11.4% for CD ≥III complications and 21.7% for CD ≤II complications (19).

The incidence of postoperative complications varies among the aforementioned studies, but the rates of major complications were generally low, which is consistent with our research findings. Active preoperative preparation and proper postoperative care measures play a crucial role in reducing the occurrence of postoperative complications. In this study, 3 patients (1.7%) experienced postoperative urinary retention. Similarly, according to the case reports registered in the 2022 CTRC database, 1.5% (29 cases) of patients also experienced postoperative urinary retention. The occurrence of urinary retention may be related to intraoperative nerve injury. Early postoperative bladder function exercises are beneficial for promoting the recovery of voiding function.

For a complex and technically challenging surgery, both intraoperative and postoperative complications are inevitably closely related to the learning curve and skill level. Even after surpassing the learning curve, the accurate identification and separation of vascular and nerve bundles play a crucial role in preserving and restoring postoperative urogenital function. Furthermore, incorrect anatomical dissection leading to entry into the wrong plane can potentially cause vascular damage (such as injury to the presacral veins or iliac vessels), resulting in intraoperative bleeding that affects the surgical field and, consequently, the surgical quality. Proper identification of the anatomical planes and proficient surgical techniques during the procedure can further reduce surgical complications and improve the surgical quality.

Anastomotic leakage is one of the common and serious postoperative complications in rectal cancer. Its occurrence can range from minor effects such as prolonged hospitalization to more severe consequences, including the need for secondary surgery or even death. Therefore, it significantly impacts the perioperative safety of patients. Multivariate analysis has shown that anastomotic leakage is an independent risk factor for distant recurrence and metastasis in patients with rectal cancer, posing a significant threat to postoperative survival outcomes (20). There is controversy regarding whether TaTME, as a bottom-up surgical approach, increases the risk of anastomotic leakage compared to conventional abdominal TME. In this study, 6.7% of patients experienced postoperative anastomotic leakage, with 2.8% classified as grade A, 3.4% classified as grade B, and 0.5% as grade C. This study comprehensively assessed the occurrence of anastomotic leakage based on postoperative clinical manifestations, abdominal drainage, abdominal imaging, and inflammatory markers, among other factors, providing more accurate data. This approach helps avoid misdiagnosis caused by insignificant clinical presentations.

In 2021, the Chinese CTRC database reported a study encompassing 1461 cases from 43 medical centers. The overall incidence of anastomotic leakage in this study was 7.0% (103/1461) (21). The updated database in 2022 reported an anastomotic leakage rate of 5.9%. Among the cases with anastomotic leakage, grade A accounted for 26.3% (30/114), grade B accounted for 37.7% (43/114), and grade C accounted for 33.3% (38/114) (13). International registry studies on TaTME have reported anastomotic leakage rates ranging from approximately 6.7% to 9.8% (18, 19). The data mentioned above are consistent with our center’s findings. It is worth noting that the anastomotic leakage in our center primarily consisted of grade A and grade B, with only one of grade C anastomotic leakage. Further analysis revealed that the international TaTME registry database had a diverting loop ileostomy rate of 88.3%. In the CTRC database of 2022, the ileostomy rate was 57.2%. However, in our center, the rate of ileostomy was as high as 98.6%, with almost all patients undergoing routine diverting loop ileostomy. No significant difference was observed in the occurrence of anastomotic leakage based on ileostomy, but there was consistent evidence of a lower incidence of grade C anastomotic leakage.

There is still uncertainty regarding the definitive protective effect of ileostomy on anastomotic leakage (22). However, its role in reducing the occurrence of severe grade C anastomotic leakage can be observed. Considering the high risk and serious consequences associated with anastomotic leakage, our center routinely performs ileostomy during surgery and closes it 3 months later, unless specifically requested by the patient or their family. Even if prophylactic ileostomy does not have a significant effect on reducing anastomotic leakage, it can at least mitigate the immense risk associated with its occurrence and the secondary trauma it imposes on the patient.

There is still some controversy regarding the effectiveness of using staplers to reduce the occurrence of anastomotic leakage after surgery. Our study found that manual anastomosis is an independent risk factor for postoperative anastomotic leakage. The incidence of anastomotic leakage was lower in patients who underwent stapled anastomosis compared to those who underwent manual anastomosis, with rates of 3.7% (5/134) and 15.6% (7/45), respectively. Data analysis by the international TaTME collaborative group has shown that manual anastomosis carries a higher risk of anastomotic leakage compared to stapled anastomosis. In the analysis of the 2019 CTRC database, which included 563 patients who underwent TaTME, a total of 43 cases (7.6%) experienced anastomotic leakage, which was the most significant postoperative complication (23). Univariate analysis was performed to select variables with a P-value of less than 0.1, and these variables were included in the multivariate analysis. The results showed that not using a stapler for anastomosis (P=0.004) and not performing prophylactic ileostomy (P=0.009) were independent risk factors for the occurrence of anastomotic leakage after laparoscopic TaTME surgery. According to the large-scale international TaTME registry studies, the utilization rate of staplers for anastomosis was found to be 66% (24). However, in analyzing the risk factors for anastomotic leakage, the study did not find a significant correlation between the use of staplers and the occurrence of anastomotic leakage.

Based on our research data and experience, the use of staplers contributes to a certain extent in reducing the incidence of anastomotic leakage. Moreover, the use of staplers is particularly beneficial as it helps to shorten the operating time in the anal region and reduces the functional damage caused by prolonged dilation of the port. From a clinical efficacy perspective, the benefits outweigh the risks. Higher quality and larger sample size prospective studies are warranted to further investigate and establish the protective role of the stapler.

## Conclusion

TaTME, as an emerging technique, is still in its early stages compared to laparoscopic TME. However, based on current research and our center’s data and experience, TaTME has shown promising perioperative outcomes. Our study identified male gender, a pathological T stage ≥ 3, and manual anastomosis as independent risk factors for postoperative major complications in a cohort of mid and low rectal cancer patients undergoing TaTME. Given the limited sample size and the sufficiently long follow-up period in this study, it is worthwhile to conduct larger, higher-quality multicenter studies in the future. Furthermore, there is limited literature available on the long-term efficacy of TaTME. Subsequent studies conducted by our research team will focus on long-term survival outcomes, utilizing our center’s data, to further validate and explore these aspects.

## Data availability statement

The original contributions presented in the study are included in the article/supplementary material. Further inquiries can be directed to the corresponding author.

## Ethics statement

The studies involving humans were approved by the Ethics Committee at the First Affiliated Hospital of Xiamen University. The studies were conducted in accordance with the local legislation and institutional requirements. Patient consent was waived due to the retrospective character of the study, and it was approved by the Ethics Committee at the First Affiliated Hospital of Xiamen University. All procedures performed in studies involving human participants were conducted according to the ethical standards of the institutional research committee and the Helsinki declaration and later revision.

## Author contributions

ZX: Formal Analysis, Writing – original draft. JZ: Data curation, Writing – review & editing. HB: Software, Writing – review & editing. LX: Methodology, Writing – review & editing. TW: Resources, Writing – review & editing. HL: Resources, Writing – review & editing. QH: Supervision, Writing – review & editing. JY: Methodology, Supervision, Writing – review & editing.
